# Etymologia: *Histoplasma capsulatum*

**DOI:** 10.3201/eid2703.ET2703

**Published:** 2021-03

**Authors:** Monika Mahajan

**Affiliations:** Post Graduate Institute of Medical Education and Research, Chandigarh, India

**Keywords:** etymologia, Histoplasma capsulatum, fungi, fungal infections, histoplasmosis, cytoplasm, capsule, histiocyte-like cells, Samuel Darling, Darling’s disease, Panama Canal, zoonoses

## *Histoplasma capsulatum *[hĭs′tə-plăz′mə kăp′sə-lā′təm]

In 1905, Samuel Taylor Darling serendipitously identified a protozoan-like microorganism in an autopsy specimen while trying to understand malaria, which was prevalent during the construction of the Panama Canal. He named this microorganism *Histoplasma capsulatum* because it invaded the cytoplasm (plasma) of histiocyte-like cells (Histo) and had a refractive halo mimicking a capsule (capsulatum), a misnomer (Figure).

**Figure:**
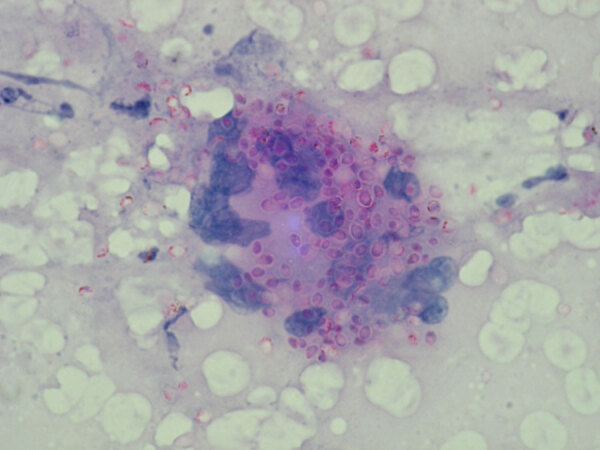
Numerous, capsulated yeast cells (shown in pink) of *Histoplasma capsulatum* in a bone marrow aspirate (Giemsa-stained, original magnification ×400). Source: Shivaprakash Rudramurthy, PGIMER, Chandigarh, India.

*Histoplasma capsulatum*, a dimorphic fungus, now belongs to Kingdom Fungi and causes histoplasmosis (Darling’s disease) through inhalation of spores found in soil and bird droppings. The fungus thrives in the central and eastern parts of United States, especially around the Ohio and Mississippi River valleys, and in South America, Africa, Asia, and Australia. Three varieties exist globally: *H. capsulatum* var. *capsulatum, H. capsulatum* var*. duboisii,* and *H. capsulatum* var. *farciminosum.*
